# Cost of Starting Colorectal Cancer Screening Programs: Results From Five Federally Funded Demonstration Programs

**Published:** 2008-03-15

**Authors:** Florence K L Tangka, Laura Seeff, Amy DeGroff, James Gardner, A Blythe Ryerson, Marion Nadel, Janet Royalty, Sujha Subramanian, Bela Bapat

**Affiliations:** Division of Cancer Prevention and Control, Centers for Disease Control and Prevention; Division of Cancer Prevention and Control, National Center for Chronic Disease Prevention and Health Promotion, Centers for Disease Control and Prevention, Atlanta, Georgia; Division of Cancer Prevention and Control, National Center for Chronic Disease Prevention and Health Promotion, Centers for Disease Control and Prevention, Atlanta, Georgia; Division of Cancer Prevention and Control, National Center for Chronic Disease Prevention and Health Promotion, Centers for Disease Control and Prevention, Atlanta, Georgia; Division of Cancer Prevention and Control, National Center for Chronic Disease Prevention and Health Promotion, Centers for Disease Control and Prevention, Atlanta, Georgia; Division of Cancer Prevention and Control, National Center for Chronic Disease Prevention and Health Promotion, Centers for Disease Control and Prevention, Atlanta, Georgia; Division of Cancer Prevention and Control, National Center for Chronic Disease Prevention and Health Promotion, Centers for Disease Control and Prevention, Atlanta, Georgia; Research Triangle Institute, Inc, Research Triangle Park, North Carolina; Research Triangle Institute, Inc, Research Triangle Park, North Carolina

## Abstract

**Introduction:**

In 2005, the Centers for Disease Control and Prevention (CDC) started a 3-year colorectal cancer screening demonstration project and funded five programs to explore the feasibility of a colorectal cancer program for the underserved U.S. population. CDC is evaluating the five programs to estimate implementation cost, identify best practices, and determine the most cost-effective approach. The objectives are to calculate start-up costs and estimate funding requirements for widespread implementation of colorectal cancer screening programs.

**Methods:**

An instrument was developed to collect data on resource use and related costs. Costs were estimated for start-up activities, including program management, database development, creation of partnerships, public education and outreach, quality assurance and professional development, and patient support. Monetary value of in-kind contributions to start-up programs was also estimated.

**Results:**

Start-up time ranged from 9 to 11 months for the five programs; costs ranged from $60,602 to $337,715. CDC funding and in-kind contributions were key resources for the program start-up activities. The budget category with the largest expenditure was labor, which on average accounted for 67% of start-up costs. The largest cost categories by activities were management (28%), database development (17%), administrative (17%), and quality assurance (12%). Other significant expenditures included public education and outreach (9%) and patient support (8%).

**Conclusion:**

To our knowledge, no previous reports detail the costs to begin a colorectal cancer screening program for the underserved population. Start-up costs were significant, an important consideration in planning and budgeting. In-kind contributions were also critical in overall program funding. Start-up costs varied by the infrastructure available and the unique design of programs. These findings can inform development of organized colorectal cancer programs.

## Introduction

Screening for colorectal cancer (CRC) reduces mortality and improves quality of life through earlier detection of precancerous polyps and thus more effective treatment of cancers (1). Less than one-half of the eligible population in the United States are up to date with recommended CRC screening tests, and the uninsured are among those least likely to participate in screening programs (2,3). Screening programs for the uninsured should help to increase the proportion of this subpopulation who are screened and improve health outcomes. Costs associated with offering screening tests and performing subsequent diagnostic procedures need to be assessed in program planning. Such economic assessment is an increasingly important tool that allows policy makers to plan for optimal allocation of limited health care resources, identify the most efficient approach to implementing screening programs, and assess annual budget implications.

CRC screening has been shown to be cost-effective in numerous studies using decision analytic models to assess the benefits and the cost of screening (4). The models have produced conflicting results on which of the screening tests for early detection of cancer recommended by the American Cancer Society guidelines (5,6) is most cost-effective. However, the models have been consistent in determining that screening for CRC with any of the recommended tests is more cost-effective than the alternative of no screening (7,8). To date, no evaluation effort has included careful assessment of the cost of offering CRC screening through organized programs in the United States. Overall costs of such programs go well beyond the cost of the individual screening tests provided. They include expenditures to hire staff, establish contracts and partnerships with providers, develop databases and other mechanisms to maintain records and track patient outcomes, recruit patients, provide professional education, and establish medical advisory boards. Programs that provide screening services to underserved populations can incur significant costs in outreach, patient education, and case management.

The Centers for Disease Control and Prevention (CDC) established the Colorectal Cancer Screening Demonstration Program (CRCSDP) in 2005 to explore the feasibility of establishing a CRC screening program for the underserved U.S. population. Data from the five program sites funded through this effort provide a unique opportunity to understand the costs associated with offering screening through organized programs. Each program is described in detail elsewhere in this issue (9,10).

CDC is undertaking a detailed evaluation of CRCSDP to estimate the cost of implementation (start-up and maintenance), describe implementation processes, assess patient outcomes, and determine the relative cost-effectiveness of screening modalities. We report here on the start-up costs of establishing CRC screening programs, which include all expenditures before delivery of the service. This information is essential for the estimation of the funding required to plan and start a CRC screening program. Subsequent reports will include costs incurred during the service delivery phase of the program.

## Methods

We developed an instrument to collect data on use of resources and costs related to the start-up of CRCSDP. The start-up period was defined as the time between the date of the funding award (August 31, 2005) and the start of delivery of the screening service in each program. We knew that the times required to complete start-up activities for each program might differ, which would lead to an inconsistent duration of the start-up period for programs. Nevertheless, we adopted this definition to ensure that start-up activities and their costs were fully captured at each program site.

**Figure 1:**
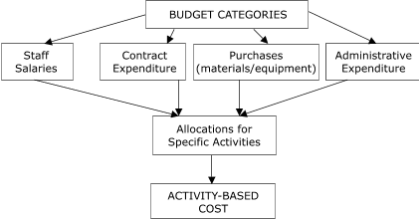
Approach to collection of cost data in study of start-up costs in five programs in Colorectal Cancer Screening Demonstration Program, 2005–2006.

Well-established methods for collecting cost data for program evaluation, such as the "ingredient approach," were considered in designing the questionnaire (11-14) (S. Subramanian, PhD, unpublished data, 2006). Costs were assigned to four budget categories: staff salaries, contract expenditure, purchases, and administrative expenditure (Figure 1), and activities were placed in these categories. Activity-based costs were derived by aggregating expenditures for staff salaries and labor, contractual costs, and purchases for each activity. We also collected overhead or indirect costs, including expenditures for items such as telecommunications and rent associated with CRCSDP. The monetary value of in-kind contributions provided to the programs during the start-up period was also estimated.

The cost instrument was designed to ensure collection of accurate data despite variations among the five programs. The programs differed in structure, provider network, selection of the screening test, and size of the service delivery area, and all of these factors affected costs. One of the five programs is implemented statewide, two are restricted to large urban areas (one city each), and two others serve clients throughout one to three counties. Two programs provide colonoscopy as the primary screening test, and the other three provide guaiac-based fecal occult blood tests. The structure for service delivery was more decentralized in some programs than in others. Decentralized programs, for example, contracted with providers to perform recruitment, screening, and patient follow-up activities, whereas centralized programs did not outsource these activities. Provider networks varied by site and included hospitals, specialty care centers, state health departments, and community health centers.

All data were collected in Microsoft Excel (Microsoft Corporation, Redmond, Washington). Program staff entered the data prospectively into the Excel work sheets to ensure accuracy of the information and avoid issues related to recall bias. For example, program staff maintained a log of the activities performed that was updated on a weekly or monthly basis. Programs were also provided with a user guide giving detailed definitions of each activity captured for the start-up period. This guide assisted with data reporting and helped to ensure consistent reporting among all programs so that meaningful comparisons could be made. Evaluators conducted a series of conference calls with each site to provide additional guidance for data collection.

We analyzed costs according to CRCSDP program activities during the start-up period. In estimating the true labor costs, we used the information collected on 1) the number of hours worked by staff per month on various activities, 2) the proportion of salary paid through CRCSDP funds, 3) data on the percentage of time staff members worked, and 4) staff salary. The staff salary information was requested as either a range or the actual base salary in addition to the fringe benefit rate. We used the average of the lower and upper bounds of the salary range when necessary. When salary information was not provided, we used national average compensation for a specific job category from the Bureau of Labor Statistics (www.bls.gov) or the average salary from a similar job category provided by the programs. On the basis of this information, we computed the hourly rate for labor and the proportion of in-kind labor cost — labor hours expended but not covered by CDC funds. The labor costs were then aggregated for each activity in each program.

For contract expenditures, we aggregated the costs of consultants and funding to provider sites by program activity, such as technology support and development of media materials. Costs for materials, equipment, and supplies were also computed for each activity in each program. We aggregated the overhead costs related to the start-up period and confirmed the activity-based cost estimates for each CRCSDP-funded activity by comparing the CRCSDP funds expended by each program. If discrepancies were noted, we contacted the program to clarify the data provided and resolve inconsistencies.

## Results

The start-up periods for the five programs ranged from 9 to 11 months. Details on the start-up activities are provided in Table 1. CDC funding and in-kind contributions were key resources for the program start-up activities. Funding sources for costs incurred by each program during the start-up period are shown in Table 2. The mean total cost for programs was $171,139. The lowest cost was $60,602, and the highest cost was $337,715.

On average, CDC funding was 42% of total cost and in-kind contribution was 50%. CDC funds ranged from 13% to 63% of total start-up costs. Two programs also received funding from other sources, including the state Comprehensive Cancer Control program and the state CRC task force. After excluding these two programs, the CRCSDP funding from CDC averaged 46%. For two of the programs, CDC funding for CRCSDP activities was substantially more than in-kind contributions. In-kind contributions varied among the programs and constituted 28% to 67% of total costs. They included donated labor time (e.g., physicians on the medical advisory board) and supplies such as computers. Labor was a major component of in-kind donations.

**Figure 2:**
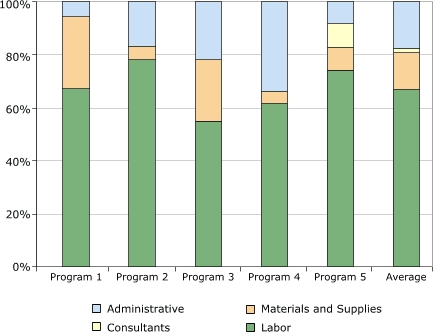
Distribution of start-up costs, by budget category, in study of five programs in Colorectal Cancer Screening Demonstration Program, 2005–2006.

Distribution of start-up costs among budget categories is shown in Figure 2. The category with the largest expenditure was labor, which on average accounted for 67% (range, 55%–78%) of the start-up cost. Only one program incurred expenditures related to consultants; these costs were 2% of the total cost among all programs. On average among the five programs, administrative cost was 17% (range, 6%–34%) of the total cost, and the cost for materials and supplies was 14% (range, 5%–27%). Materials and supplies included items such as postage, forms, brochures, and medical supplies.

**Table:** 

Numbers do not add up to 100% due to rounding.

**Figure3:**
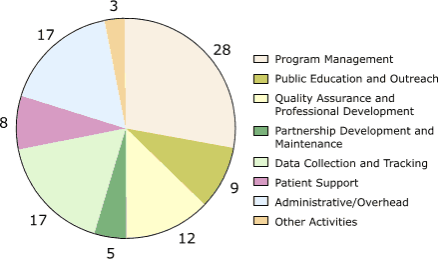
Percentage distribution of start-up costs, by activity, averaged across the five programs in the Colorectal Cancer Screening Demonstration Program, 2005–2006. Numbers do not add up to 100% due to rounding.

For all programs combined, the largest cost categories by activity were management (28%; range, 18%–34%); data collection and tracking, which were mainly for database development (17%; range, 8%–35%); administrative costs (17%; range, 6%–34%); and quality assurance (12%; range, 10%–15%) (Figure 3). Other activities with significant expenditures included public education and outreach (9%; range, 6%–13%) and patient support (8%; range, 0%–19%).

**Table:** 

Program management	28%
Public education and outreach	9%
Quality assurance and professional development	12%
Partnership development and maintenance	5%
Data collection and tracking	7%
Patient support	8%
Administrative/overhead	17%
Other activities	3%

**Figure4:**
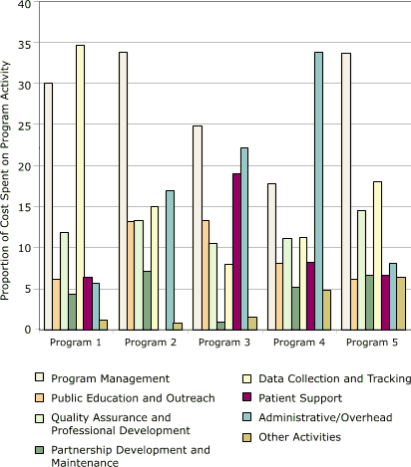
Distribution of start-up costs, by activity, in study of five programs in Colorectal Cancer Screening Demonstration Program, 2005–2006.

Cost allocations among activities for each of the five programs are shown in Figure 4. This distribution varied among the programs. Database development was the largest cost category for program 1 (35%), and administration/overhead was the largest category for program 4 (34%). The largest category for the other three programs was program management (25%–34%).

**Table:** 

Program 5 numbers do not add up to 100% due to rounding.

## Discussion

To our knowledge, no previous evaluation has provided details on the costs to begin a CRC screening program for the underserved population. The important contributions of this paper include 1) the imputation of market value to in-kind contributions (voluntary and donated services and products), which can help other programs explicitly account for in-kind contributions in their budgets in cases when these resources might not be freely available; 2) the categorization of cost components (Table 1), which provides a useful guide about different sources of costs; and 3) the provision of steps involved in the valuation of labor. Our analysis shows that start-up costs can be significant and should be considered in planning and budgeting for future CRC screening programs.

One of the largest cost components was overall program management, which involved a wide range of activities. Necessary resources included expenditures to develop fiscal systems, recruit and train staff, establish policies and procedures, and negotiate contracts with providers. Another significant cost component was developing a database system to monitor and track patient services. Labor required to perform these activities was the most significant budget category; materials and supplies accounted for a much smaller proportion of the total cost. Most of these activities represent fixed costs and therefore will not vary in relation to the volume of screens performed.

In this CRC screening demonstration, in-kind contributions were critical in providing the resources required for the start-up program activities. Therefore, total cost of start-up activities should include the monetary value of these contributions, which are generally related to donation of labor hours. This in-kind labor was mainly provided by senior management staff members, who were vital for the overall success of the program, and by physicians and other key individuals who participated in the medical advisory board to ensure that the program was designed to include pertinent clinical care partners and that quality care was provided to program participants. In future planning for resource allocation for a similar program, these critical categories should not be overlooked.

Start-up costs varied substantially across the five programs in this screening demonstration. The infrastructure available before the start of this effort accounts for some of the difference. For example, programs that could easily manipulate existing data-collection tools did not have to incur large expenses to create new data systems. Therefore, we can expect programs that plan to build on a well-established infrastructure to incur smaller start-up costs than programs with limited infrastructure. Other sources of variation could include the type of screening tests offered, the geographic area covered, the setting in which the program was created (e.g., academic medical center vs state health department), and individual program contributions to administrative costs, which were a significant proportion of total costs. Although we provided a user's guide to minimize inconsistencies in reporting data, programs may have differed in the allocation of expenses to the various activity-based categories. Systematic quantitative assessment of these variations in start-up cost was not possible because of the small number of pilot sites available. Finally, we did not assess the cost incurred in the start-up period in relation to the effectiveness of the services provided by the programs. An evaluation of the cost-effectiveness of the programs is planned, and results based on intermediate outcomes (cost per screen performed and cost per polyp/cancer detected) will be reported in future publications. However, such a study cannot be conducted in the start-up phase of a program when no screening has occurred.

The information provided in this assessment on the magnitude of cost related to specific start-up activities can serve as a guide to estimation of start-up costs for funding agencies and organizations implementing screening programs. The detailed list of start-up activities developed for this study can also assist program staff to develop budget estimates, and the real-world cost values reported in this analysis can serve as a benchmark for evaluation of these estimates. Thus, our work should provide essential information for the successful start of CRC screening programs. Furthermore, details on implementation costs are expected to provide in-depth assessment of the costs associated with recommended screening tests and realistic estimates of the cost of diagnosis and complications.

## Figures and Tables

**Table 1:** Activity-Based Categories in Study of Start-Up Costs in Five Programs in Colorectal Cancer Screening Demonstration Program, 2005–2006

Program Activity	Description
Program management	Defining specific measurable and realistic objectives, including goals of screening Recruiting, hiring, and training staff Developing fiscal system Collaborating with Centers for Disease Control and Prevention Establishing and managing related contracts Identifying and contracting with local physicians and clinics to deliver screening services Developing administrative policies and procedures Managing programmatic, administrative, and reporting issues Traveling for program meetings Establishing necessary administrative billing and reimbursement system
Public education and outreach	Developing and planning public education and outreach activities Conducting outreach and in-reach activities Conducting and facilitating related training Collaborating with partners
Quality assurance and professional development	Convening medical advisory board Developing quality-control standards and mechanisms Developing clinical policies and procedures Developing or enhancing training to educate and train health care professionals
Partnership development and maintenance	Developing and maintaining partnerships (e.g., Comprehensive Cancer Control, medical health care systems, businesses)
Data collection and tracking	Developing and adapting data-collection and reporting system Establishing surveillance system to track clients with abnormal screening results or diagnosis of cancer and follow up with them
Patient support	Establishing patient support system to provide appropriate screening, diagnostic, and treatment services Planning and identifying funding sources to ensure treatment services for people with cancer diagnosis or medical complications
Other activities	Designing plan for program evaluation, job orientation, and training for collection of data on cost
Administrative/overhead costs	Indirect costs (e.g., rent, telecommunications, maintenance)

**Table 2:** Distribution of Start-Up Costs, by Funding Source, Study of Five Programs in Colorectal Cancer Screening Demonstration Program (CRCSDP), 2005–2006

Funding Source	Programs					Mean

	1	2	3	4	5	
Total, $	145,410	60,602	146,193	337,715	165,775	171,139
In-kind contributions,^a^ %	63	58	37	67	28	50
CDC funding, %	13	42	63	33	57	42
Other,^b^ %	24	0	0	0	15	8

All percentages are computed as percentage of the total cost.

a Labor costs and nonlabor costs (e.g., telephone calls, bowel preparation kits, printing of data-collection forms).

b Includes Comprehensive Cancer Control program and colorectal cancer task force funding.
